# Biological treatments for pediatric Netherton syndrome

**DOI:** 10.3389/fped.2022.1074243

**Published:** 2022-12-23

**Authors:** Matteo Pontone, Mattia Giovannini, Cesare Filippeschi, Teresa Oranges, Fausto Andrea Pedaci, Francesca Mori, Simona Barni, Federica Barbati, Filippo Consonni, Giuseppe Indolfi, Lorenzo Lodi, Chiara Azzari, Silvia Ricci, Alain Hovnanian

**Affiliations:** ^1^ Department of Health Sciences, University of Florence, Florence, Italy; ^2^ Allergy Unit, Department of Pediatrics, Meyer Children's Hospital, Florence, Italy; ^3^ Dermatology Unit, Department of Pediatrics, Meyer Children's Hospital, Florence, Italy; ^4^ Pediatric and Liver Unit, Meyer Children's University Hospital, Florence, Italy; ^5^ Immunology Unit, Department of Pediatrics, Meyer Children's Hospital, Florence, Italy; ^6^ INSERM UMR 1163, Laboratory of Genetic Skin Diseases, Imagine Institute and University of Paris, Paris, France; ^7^ Department of Genetics, Necker Hospital for Sick Children, Assistance Publique des Hôpitaux de Paris (AP-HP), Paris, France

**Keywords:** Netherton syndrome, biological treatments, immunology, dermatology, allergology, pediatrics

## Abstract

Netherton syndrome (NS) is a rare and potentially life-threatening genetic skin disease responsible for skin inflammation and scaling, hair abnormalities and severe allergic manifestations. NS is caused by loss-of-function variants in Serine Peptidase Inhibitor Kazal-Type 5 (*SPINK5*)*,* encoding the serine protease inhibitor LEKTI. NS patients have a profound skin barrier defect caused by unopposed kallikrein-related proteases activity (KLKs). They develop severe skin inflammation with eczematous-like lesions and high serum IgE levels. Multiomics studies have revealed that the IL-17/IL-36 pathway is the most predominant upregulated pathway in NS. It is associated with a Th2 signature with complement activation in the ichthyosis linearis circumflexa subtype, and with interferon and Th9 activation in the scaly erythrodermic form. Several case reports proved the efficacy of different biotherapies targeting IL-17A, IL-12/IL-23, IL-4R and IL-13R, TNF-a and IL-1β in pediatric NS patients. Intravenous immunoglobulins (IVIG) have also shown efficacy. These studies showed no severe side effects. At present, IL-17 blockade seems to be the most efficient treatment, but case reports remain limited with small numbers of patients and no placebo-control. Additional pathways must also be explored, and more efficient strategies could be used to block IL-17 and IL-23 pathways. In the future, the combination of specific strategies aiming at repairing the initial skin barrier defect could potentiate the efficacy of biologics. The current reports suggest that biological therapy is safe and often effective at pediatric age. However, controlled clinical trials that include a larger number of patients need to be conducted to reach more reliable conclusions.

## Introduction

NS is an autosomal recessive disease characterized by congenital ichthyosiform erythroderma, hair shaft abnormalities and atopic manifestations (1). It causes life-threatening complications in the neonatal period and in infancy, e.g., due to severe dehydration, systemic infections and failure to thrive. NS presents most often as congenital scaly erythroderma (SE), which can persist during childhood or evolve into ichthyosis linearis circumflexa (ILC) (2). Specific hair shaft abnormality, referred to as trichorrhexis invaginata (or bamboo hair), is responsible for hair fragility and partial alopecia. Eczematous-like lesions, severe pruritus, upper airway infections and multiple food allergies with high serum IgE levels are almost constant features. NS evolves by flare-ups with lesions likely to be infected.

NS results from loss-of-function mutations in the *SPINK5* gene encoding the serine protease inhibitor Lympho-Epithelial Kazal-Type-related protease Inhibitor (LEKTI). LEKTI is normally expressed in the granular layer of the epidermis and inhibits kallikrein-related serine proteases (KLK) through pH-dependent chelation. The extracellular pH decreases from the granular layer to the stratum corneum where LEKTI dissociates from its targets, allowing the skin to desquamate through KLKs’activity (2).

NS patients suffer from LEKTI deficiency and KLKs'proteolytic excessive activity. Uncontrolled KLK cause early desquamation of the epidermis and degrade hair follicle proteins. Overactive KLK also contribute to the skin barrier defect by increasing filaggrin degradation and impairing the lipid composition of the stratum corneum (3, 4). KLK also activate Protease-Activated Receptor 2 (PAR-2) causing downregulation of lamellar bodies secretion in the stratum corneum (5). Overall, the unopposed activity of these KLK leads to profound alterations of the skin barrier and increased epithelial vulnerability (2).

The skin barrier disruption facilitates pathogen penetration, which drives a TH17 immune response through the production of pro-inflammatory cytokines (IL-6, IL-17C, IL-36) (2, 6, 7).

In parallel, KLK hyperactivity triggers TH2 immune response (2). Therefore, NS shares common features with atopic dermatitis (TH2 immune response) and others with psoriasis (increased levels of IL-36, IL-23 and IL-17) (2, 8, 9, 10).

Studies have shed light on the cytokine-mediated pathogenesis of NS individuating therapeutic targets for specific biologic drugs. This article aims to summarize the biological treatments used in the management of pediatric NS patients (less than 18 years of age) and to discuss potential future biotherapies and their place in NS treatment.

## Case reports for biological treatments for pediatric NS

### IL-17A targeting

NS patients show increased IL-17A signaling (6). Secukinumab is a human antibody capable of binding IL-17A, thus preventing interaction with its receptor.

Luchsinger et al*.* (11) described the compassionate use of subcutaneous (SC) secukinumab in 4 patients; two of them were of pediatric age and presented with an ichthyosiform erythroderma. Secukinumab was weight-adjusted: 75 mg for less than 25 kg, 150 mg for 25 to 50 kg, and 300 mg for greater than 50 kg at baseline and at weeks 1, 2, 3, 4, and monthly thereafter. Patients were followed for 1 year, during which they continued skincare routine and used topical steroid therapy only in the case of exacerbation. Patients were evaluated at 3 and 6 months after treatment initiation and the treatment response was assessed by Ichthyosis Area and Severity Index (IASI), Dermatology Life Quality Index (DLQI), Children's DLQI and 5-D itch scale. At 3 and 6 months, the authors reported decreasing in the scores mentioned above, with the best improvement seen in the two pediatric patients. The authors described the reduction in pruritus and in the use of topical steroids; the two pediatric patients showed improvement in growth rate at 6th month evaluation.

Blanchard et al*.* (12) reported a case of a 16-year-old male presenting with erythematous scaly plaques on the face, abdomen and extremities. Authors administered SC secukinumab 300 mg weekly for 4 weeks, and then 300 mg monthly. After 4 weeks of therapy, facial erythema was remarkably improved and healed after 3 years of treatment. Gan et al*.* (13) described the treatment with SC secukinumab of 2 pediatric patients: the younger (newborn) presented with ichthyosiform erythroderma and received 150 mg of secukinumab weekly for 5 weeks, then monthly. The older (suckling) presented with ILC and received 75 mg of Secukinumab weekly for 4 weeks, then monthly. Both patients showed improvement of skin conditions and decrease in flare frequency and duration: the first patient after 4 weeks of therapy during a 6 month follow-up, the second after 3 months of therapy during a 12 months follow-up. None of them experienced adverse reactions to secukinumab.

### IL-12 and IL-23 targeting

The IL-12*β*1 receptor is expressed on the surface of keratinocytes and is activated by IL-12 and IL-23. Binding of IL-12 leads to a TH1 immune response, while IL-23 triggers a TH17 immune response. Ustekinumab is a human antibody binding the p40 subunit shared by IL-12 and IL-23, preventing their interaction with the IL-12*β*1 receptor.

Volc et al*.* (14) reported treatment with ustekinumab in a 15-year-old female patient presenting with generalized fine scaling and polycyclic plaques on the trunk and lower extremities without disease control. Due to clinical similarities between the patient's lesion and psoriasis, the authors decided to start SC ustekinumab 45 mg (0.75 mg/kg) at baseline and at week 4 and every 12 weeks thereafter. They described improvement of cutaneous manifestations after the second dose of ustekinumab and a persistent remission after one year of therapy.

### IL-4R**α** and IL-13 targeting

IL-4 and IL-13 are key actors of the TH2 response described in NS. Dupilumab targets the IL-4 receptor subunit alpha, which is a component of both the IL-4 and IL-13 receptors, so that dupilumab will inhibit signaling regulated by the aforementioned cytokines.

Süßmuth et al*.* (15) reported the use of dupilumab in 2 pediatric patients. The first patient was a 12-year-old female treated with 600 mg followed by 300 mg administered subcutaneously every 4 weeks; after 4 months, the therapy was intensified to 200 mg biweekly for 8 more months. The second patient was an 8-year-old male treated with 300 mg every 4 weeks for 10 months administered subcutaneously; this patient had been previously treated with weekly SC immunoglobulins (SCIG), and he was still under Ig during dupilumab treatment. The response to dupilumab was assessed by Netherton Area Severity Assessment (NASA) and Physician Global Assessment (PGA), and changes in pruritus intensity were evaluated by a numeric rating scale (NRS). The authors described an improvement in NASA, PGA and NRS in the 2 patients during dupilumab treatment (by the 4th month of therapy in the first patient, by the 10th month of therapy in the second one); in the male patient, they even managed to reduce Ig from weekly administration to biweekly administration. The authors also reported a decrease in IgE and eosinophils for 1 patient and a decrease in IgE for the other patient. The authors reported significant difference among inflammatory cytokines (IL-1β, IL-6, IL-17A, IL-1RA, IL-18, CXCL9) measured before and after treatment.

Murase et al*.* (16) reported treatment of 2 patients with dupilumab; 1 patient was a 17-year-old female presenting with erythroderma of the face and trunk and ichthyosis linearis circumflexa on the extremities. The authors started dupilumab 600 mg at baseline and then 300 mg every 2 weeks. After 6 months of therapy, Clinical Ichthyosis Score (CIS), Eczema Area and Severity Index (EASI), Investigator Global Assessment (IGA), and Visual Analogue Scale (VAS) for itching were improved compared to scores registered before dupilumab treatment. Serum IgE and thymus- and activation-regulated chemokine (TARC) levels decreased during dupilumab therapy, while IL-4 and soluble IL-2 receptor levels did not change significantly. Skin lesions and hair volume and length were improved after respectively 10 months and 6 months of dupilumab treatment.

### TNF-α targeting

Increased TNF-α levels have been reported in NS. Infliximab is a chimeric antibody targeting both circulating TNF-α and transmembrane TNF-α. Cicek et al*.* (17) described the pediatric use of infliximab. The patient presented with recurrent sepsis, eczematous dermatitis and scaling from birth, and showed increased serum IgE levels and eosinophil count. The authors started intravenous (IV) infliximab infusions at 6 months of age, at a dosage of 5 mg/kg at baseline and at weeks 2, 6 and every 4 weeks thereafter. Complete blood cell count and kidney and liver function tests were carried out every month, with no abnormalities reported. After the third infusion of infliximab, the authors observed a decrease in skin severity, and after one year of therapy, the skin healed, allowing the discontinued use of infliximab therapy.

### IL-1β targeting

The IL-1β pathway is strongly upregulated in NS. Anakinra is a competitive antagonist of IL-1*α* and IL-1β through the binding of IL-1 type I receptor (IL-1RI). Ragamin et al*.* (18) reported the treatment of a 7-year-old female with anakinra. The patient presented with ILC and erythroderma. The authors administered 100 mg of SC anakinra daily for 5 months, observing improvement of skin lesions and increased patient strength and social activities after 1 month of therapy. During treatment, no changes in laboratory values were registered. Therapy was stopped due to the patient's scarce compliance to injection, and NS rapidly worsened.

## Immunoglobulin replacement therapy

NS has long been considered a primary immunodeficiency syndrome (19) which led to the use of immunoglobulin replacement therapy (IRT) to treat NS children.

The use of IRT may improve NS course because of the capability of Ig to opsonize bacteria, stimulating phagocytosis and reducing intercurrent infection frequency and inflammation. At the same time, IVIG may cause aseptic meningitis, anemia, leukopenia and thromboembolic events (20).

Small et al*.* (21) reported treatment of a 16-year-old female and a 10-year-old male presenting with pustular lesions, similar to psoriasis, and ichthyosis linearis circumflexa. Patients were treated with IVIG at 500 mg/kg monthly for 3 months, and after 3 doses of IVIG, patients'symptoms (pruritus) and signs (erythema, pustulation, scale) improved.

Zhang et al*.* (22) described the case of a 3-year-old male presenting with generalized erythroderma scaly skin and ichthyosis linearis circumflexa. The patient was first diagnosed with atopic dermatitis and treated with topical steroids and 2 doses of IVIG (1 g/kg). Once NS was diagnosed, the authors started IVIG 500 mg/kg monthly for 2 months with a remarkable improvement in skin lesions by the end of the treatment.

Zelieskova et al*.* (23) reported the treatment of a 2-year-old male presenting with generalized exfoliative erythroderma, ichthyosiform dermatitis, trichorrhexis invaginata, hypernatremic dehydration, failure to thrive, and recurrent respiratory infections. Monthly IVIG therapy was administered from 4 months of age until 12 months when – due to poor venous access – the authors switched to 1 g every 2 weeks (200 mg/kg/month) of SCIG. They recorded a progressive improvement of erythroderma and ichthyosis with the treatment, accompanied by weight gain and reduction in respiratory morbidity.

Gallagher et al*.* (24) administered monthly IVIG to a 4-month-old female presenting with a rash and failure to thrive. Then, they switched to weekly SCIG for 47 weeks due to difficult venous access. The authors describe an improvement of growth - weight from lower then 3th percentile to 10–25th percentile and height from below 3th percentile to 3–5th percentile – and of skin symptoms – a decrease of pruritus and ichthyosis gravity – observed after 8 months of therapy. Renner et al*.* (19) reported 9 pediatric patients with NS (Table 1). All patients presented with the classical NS triad – congenital ichthyosis, bamboo hair and allergic diathesis. One patient lacked bamboo hair. All patients revealed eosinophilia and increased serum IgE levels. Five of the 9 patients were treated with a dosage of 400 mg/kg of IVIG monthly for 2 years due to abnormal antibody responses to bacteriophages (4 patients) or failure to thrive (1 patient). Treated patients showed improved skin clinical manifestations and growth curves.

**Table 1:** Main aspects of pediatric case studies mentioned in the text.

Reference	Patients		SPINK5 mutation	Presentation	Therapy					
	Sex	Age (years)			Type	Duration (at study time)	Outcome		Side effects
							Response to therapy	Time to response	
Luchsinger *et al.* (11)	M	9	Heterozygous c.153delT (*p*.Gln52Lysfs*6) (exon 3) and c.891C > T (*p*.Cys297Cys) (exon 11)	SE	Secukinumab SC: 75 mg for less than 25 kg, 150 mg for 25 to 50 kg, and 300 mg for greater than 50 kg at baseline and weeks 1, 2, 3, and 4 and monthly thereafter	8 months	Decrease in IASI, DLQI, Children's DLQI, 5-D itch scale	3 months	acute pruritic palmoplantar eczematous reaction	
							Improvement of growth rate	6 months		
	M	9	Homozygous c.1431-12G > A (intron 15)	SE		7 months	Decrease in IASI, DLQI, Children's DLQI, 5-D itch scale	3 months	acute pruritic palmoplantar eczematous	
							Improvement of growth rate	6 months		
Blanchard *et al.* (12)	M	16	–	SE	Secukinumab SC: 300 mg/week for 4 weeks; 300 mg/month thereafter	3 years	Improvement of facial and trunk rash	4 weeks	–	
							Clearance of facial rash	3 years		
Gan *et al.* (13)	M	<1	–	SE	Secukinumab SC: 150 mg/week for 5 weeks; 150 mg/month thereafter	12 months	Improvement in skin shedding, scaling and erythema	4 weeks	–	
	M	<1	–	ILC	Secukinumab SC: 75 mg/week for 4 weeks; 75 mg/month thereafter	6 months	Decrease in erythema and scaling	3 months	–	
Volc *et al.* (14)	F	15	Homozygous c.1431-12G > A (intron 15) and c2472_2473delAG (exon 26)	SE	Ustekinumab SC: 45 mg at baseline and week 4; every 12 weeks thereafter	12 months	Substantial improvement of skin symptoms	2 weeks	–	
Süßmuth *et al.* (15)	F	12	–	SE	Dupilumab SC: 600 mg at baseline, 300 mg every 4 weeks thereafter; after 4 months, 200 mg every 2 weeks	12 months	Decrease in NASA, PGA and NRS	4 months	Bacterial superinfection	
	M	8	–	SE	Dupilumab SC: 300 mg every 4 weeks	10 months	Decrease in NASA, PGA and NRS	Within 10 months of therapy	–	
Murase *et al.* (16)	F	17	Homozygous c.2368C > T (*p*.Arg790*)	SE and ILC	Dupilumab SC: 600 mg at baseline, 300 mg every 2 weeks thereafter	6 months	Decrease in CIS, EASI, IGA, VAS	6 months	–	
Cicek *et al.* (17)	M	<1	Homozygous c.410 + 1G > A	SE	Infliximab IV: 5 mg/kg at baseline and weeks 2, 6; every 4 weeks thereafter	12 months	Resolution of skin and scalp lesion	12 months	–	
Ragamin *et al,* (18)	F	7	–	SE and ILC	Anakinra SC: 100 mg/day for 5 months	5 months	Improvement of erythroderma and scaling on the face	1 month	–	
Small *et al.* (21)	F	16	–	ILC	IVIG: 500 mg/kg monthly	3 months	Decrease in erythema, pustulation, scale, and pruritus	3 months	–	
	M	10	–	ILC		3 months	Decrease of erythema, pustulation, scale, and pruritus	3 months	–	
Zhang *et al.* (22)	M	3	Heterozygous c.80A > G (*p*.Gln27Arg ) and deletion (chr5:147444834-147445034) (exon 2)	SE	IVIG: 500 mg/kg monthly	2 months	Improvement of skin rash	2 months	–	
Zelieskova *et al.* (23)	M	2	Homozygous c.1530CA (*p*.Cys510*)	SE	IVIG monthly; SCIG 200 mg/kg monthly thereafter	–	Decrease in erythroderma and ichthyosis	–	–	
Gallagher *et al.* (24)	F	<1	–	SE	IVIG monthly; SCIG weekly thereafter	47 weeks	Improvement of growth rate Improvement of skin lesions	8 months	Urinary tract infection Mild swelling at injection site	
Renner *et al.* (19)	M	6	Homozygous c.2459-2468delA K823RfsX100 (exon 26)	CI	IVIG 400 mg/kg monthly	2 years	Improvement of growth rate Improvement of skin lesions	2 years	–	
	M	9	Heterozygous IVS15 + 13G > A10 and not determined	CI		2 years	Improvement of skin lesions	–	–	
	M	<1	Heterozygous c.377-8delAT Y126X (exon 5) and c.2473-4delGA E825GfsX1 (exon 26)	CI		2 years	Improvement of growth rate Improvement of skin lesions	1 year	–	
	M	7	Homozygous c.2459-2468delA K823RfsX100 (exon 26)	CI		2 years	Improvement of growth rate Improvement of skin lesions	6 months	–	
	M	6	–	CI		2 years	Improvement of skin lesions	–	–	
Dabas *et al.* (25)	F	12	Homozygous deletion (chr5:147499882-147499885) (exon 26)	SE	IVIG 400 mg/kg monthly	6 months	Reduction in erythema and scaling	Within 6 months of therapy	thrombosis of left sigmoid, and transverse sinus

SE, scaling erythroderma; ILC, ichthyosis linearis circumflexa; CI, congenital ichthyosis.

Dabas et al*.* (25) observed a 12-year-old patient presenting with erythroderma who received monthly IVIG at the dosage of 0.4 g/kg for 6 months. The authors reported improvement in the skin signs and symptoms by the end of the treatment. However, the patient experienced thrombosis of the left sigmoid and transverse sinus, leading authors to stop the IVIG treatment.

None of the previous studies evaluated cutaneous signs and symptoms through defined scoring systems, but rather through a coarse visual estimation, which represents a limitation of these reports.

## Future perspectives

Biological drugs have also shown efficacy in adult NS patients (7, 18, 26, 27) suggesting that additional biotherapies might be suitable for pediatric patients as well in the next years (Table 2).

**Table 2:** Main aspects of adult case studies mentioned in the text.

References	Patients		SPINK5 mutation	Presentation	Therapy
	Sex	Age (years)			
Yalcin *et al.* (26)	M	20	–	SE	Omalizumab SC: 400 mg/kg
Barbieux *et al.* (7)	F	29	c.880_882del and c.1820 + 2T > A	ILC	Ixekizumab SC: 160 mg at baseline, then 80 mg bi-monthly for 12 weeks, then 80 mg monthly for 12 weeks
	M	30	c.238dup	SE	
	F	20	c.55 + 1G > A and c.2015 + 5G > A	ILC	

SE, scaling erythroderma; ILC, ichthyosis linearis circumflexa; CI, congenital ichthyosis.

Barbieux et al*.* (7) treated 3 adult NS patients with Ixekizumab for 6 months. Ixekizumab is a humanized antibody targeting IL-17A, acting in the same way as secukinumab. The patients showed improved pruritus and skin clinical manifestations during the induction phase. Interestingly, during the maintenance phase, clinical benefits persisted in the 2 patients presenting with ILC, while pruritus worsened in the patient with SE.

Ragamin et al*.* (18) treated 2 young adult patients with Ixekizumab. Both patients showed improvement in skin lesions, but one of the two experienced a progressive decrease in drug effectiveness after 1.5 years and this led to stop therapy after 1.9 years.

Yalcin et al*.* (26) reported the use of omalizumab in an adult NS patient. Omalizumab is a human antibody targeting Ig-E and preventing its binding to high-affinity Fc*ε*RI receptors and low-affinity Fc*ε*RII receptors. The authors described improved skin clinical manifestations and decreased markers of inflammation after 4 months of therapy.

A major difficulty is to identify the biological pathway whose inhibition will be the most effective for blocking the pathogenic mechanism of the disease. Recent reports indicate that the IL-36 and IL-17 pathways are the most upregulated pathways in both clinical NS subtypes, pointing to these biological cascades as major therapeutic targets.

Importantly, Barbieux et al*.* (10) have recently demonstrated that although the IL-17/IL-36 pathways predominate in both clinical subtypes, the immune signature differs between NS patients presenting with ILC and SE: ILC-NS patients show a Th-2/complement driven immune response, while SE-NS patients show a Th9/type I interferon driven immune response. These findings may lead to individualized biological therapies for NS based on their clinical presentation and immune signature.

It should be noticed that these multiomics studies involved mainly adults and that it is not yet known to what extent pediatric NS resembles adult NS.

The advances in the knowledge of the cytokine-mediated pathogenesis of NS and the development of new biologics open the possibility of targeting additional immunological actors, some of which may be major disease determinants. These include targeting the IL-36 pathway using the recently described IL-36R inhibitor spesolimab; blocking the IL-17R common to IL-17 (A, C and F) with brodalumab which may prove to be more efficient than blocking IL-17A alone; and inhibiting specifically IL-23p19 (risankizumab, guselkumab, tildrakizumab) which would block both Th17 and Th22 pathways; inhibiting IL-31 (nemolizumab) inhibiting the Th2 pathway. In the light of the results of the multiomics studies in the two major clinical forms of NS, it is likely that NS patients should be stratified and treated according to their specific clinical subtypes and their immune profiles. Specifically, biotherapies targeting the complement and Th2 responses could be used in NS-ILC patients, whereas other drugs, such as JAK inhibitors would be more appropriate in NS-SE patients to block the IFN pathway.

For these reasons, multiomics studies are warranted to explore these disease pathways in pediatric NS patients and confirm their rationale in children.

In the future, the development of specific inhibitors of KLK5, KLK7 and/or KLK14 could open the possibility of blocking very early disease events responsible for the profound skin barrier defect. Their combination with other drugs/biologics could increase their efficacy in the presence of a repaired skin barrier.

## Discussion

All biological therapy reports for NS paediatric patients have been limited case reports, with no placebo control, describing a total of 20 patients in 13 studies (1 to 4 patients per study). Among different reports, authors have administered different dosage of drug for different periods of time. Treatment response has not been evenly evaluated, with some reports using appropriate scoring systems and some others just a subjective clinical evaluation depending on the author experience. In addition, the multiplicity of targeted inflammation or allergy pathways prevents to draw any formal conclusion, although among them, blocking IL-17 consistently led to clinical benefit in all 5 pediatric patients treated. Although these studies remain small, they seem to show that biological therapy is suitable for NS patients. In addition, they were limited in time and long-term efficacy and tolerance remain unknown for many of them. It is likely that early treatment and patient stratification based on immune profile and clinical subtype could improve biologics efficacy.

The latest International Union of Immunological Societies (IUIS) classification of inborn errors of immunity (28) includes NS in the hyper IgE syndromes (HIES) group, among combined immunodeficiencies with associated or syndromic features. One of the main issues associated with biological therapy concern its effect on the immune system and the possibility of intercurrent infections. IL-17A plays a major role in host defence against fungi and bacteria, with still an unclear role against viruses (29). Blocking IL-17A with secukinumab or ixekizumab may increase the risk of infections, specially involving respiratory airways (upper and lower), otitis, oral herpes, tinea pedis and candidiasis (30). IL-12 bridges innate immunity and adaptive immunity through differentiation of CD4 T cells to Th1 cells, acting a major role in host defence against pathogens (31); IL-23 stimulates Th17 cells to produce IL-17 (31). Blocking IL-12 and IL-23 with ustekinumab may increase the risk of airways infections, cellulitis, dental infections, herpes zoster, genital mycosis (32). IL-4 and IL-13 are among main cytokines involved in Th2 driven immune response (33, 34); blocking IL-4 and IL-13 with dupilumab may cause increased risk of eye infections (conjuntivitis, blepharitis, cheratitis) and oral herpes (35). IL-1β is a potent proinflammatory mediator, involved in both innate immune response and adaptive immune response (36); given its wide role in immune response, blocking IL-1β with anakinra may increase risk of infections and infestations (37). TNF-α is a pivotal cytokine in phagocyte activation and granuloma formation (38). Blocking TNF-α with infliximab lead to increase of serious viral and bacterial infections and less commonly to fungal infections or tuberculosis (39). Among articles of pediatric interest, some authors (11, 15) reported infectious episodes - bacterial, fungal and viral - during treatment; infection was managed with specific therapies and none led to NS biological therapy interruption. In the case reports analysed, only Luchsinger et al*.* (11) described an adverse reaction occurring during secukinumab treatment, presenting as an acute pruritic palmoplantar eczematous reaction refractory to steroid therapy. The authors interpreted it as a psoriasiform palmoplantar reaction described in patients treated with secukinumab and it did not limit patient therapy. An important aspect is that it is now considered that NS patients show no evidence of immune deficiency (40). Therefore, biotherapies that impair the immune defence should not expose NS patients to a risk of infections significantly higher than other conditions with no immune deficiency. Nevertheless, close monitoring of patients subjected to biotherapies should be considered to promptly diagnose infectious complications, to start anti-microbial prophylaxis if necessary and to plan adequate vaccination strategy prior and during biological treatment.

**Figure 1:**
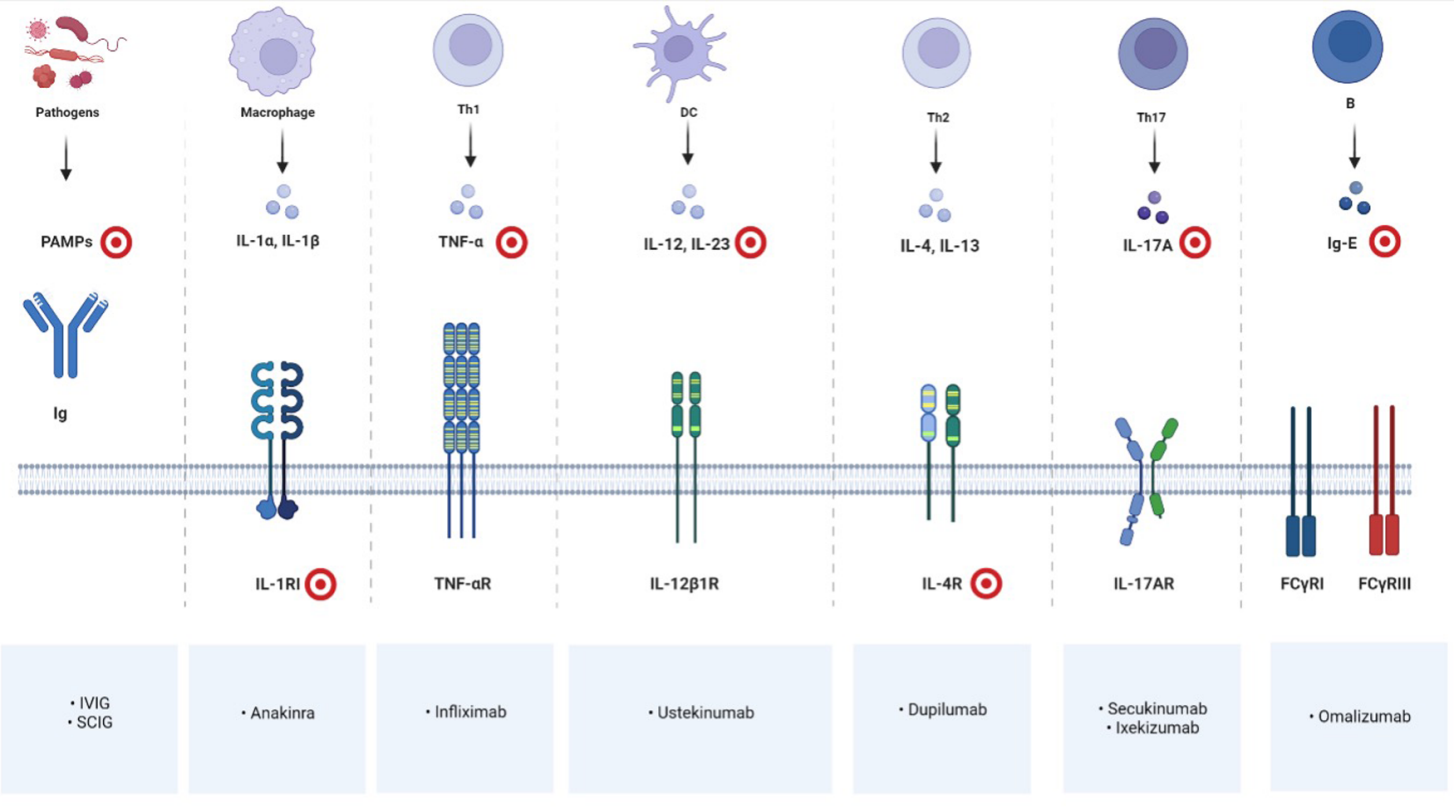
Immune cells, mediators and receptors involved in NS and their biological inhibitors; red and white targets showing drug'sites of action. Created with BioRender.com.

Our literature review suggests that biological therapy for patients with NS is safe and often effective at pediatric age, although controlled clinical trials incorporating a larger number of patients need to be conducted to draw more reliable conclusions. On the basis of reported cases, some recommendations could be made. IVIG is suggested to treat recurrent and severe infections with severe failure to grow in infants and in children until growth becomes normal and infections stop. In the case of a very inflammatory and itchy form of scaly erythroderma or ILC, a biotherapy blocking IL-17A (such as Secukinumab or Ixekizumab) appears to be currently the best therapeutic option to improve the permanent inflammatory condition of the skin and/or to prevent acute flares. Figure 1 resumes targets of immunoglobulin replacement therapy and of current available biotherapies for pediatric NS.

## Conclusion

We summarized information generated from case reports published on the treatment of paediatric NS patients with biological therapy. More data need to be collected from upcoming clinical trials to define therapeutic strategies and care plans for NS patients and to describe possible phenotype/genotype correlation regarding treatment response. In the presence of recurrent and severe infections with severe failure to grow, IVIG is recommended in infants and in children. In the case of very inflammatory and itchy form of scaly erythroderma, a biotherapy blocking IL-17A (such as Secukinumab or Ixekizumab) appears to be currently the best therapeutic option according to several case reports.

Individual profiling of the type of immune response opens possibilities of using biologics to target specific biological pathways for precision medicine.
